# RETRACTION: LncRNA SNHG14 Contributes to the Progression of NSCLC Through miR‐206/G6PD Pathway

**DOI:** 10.1111/1759-7714.70154

**Published:** 2025-08-26

## Abstract

**RETRACTION**: ZhaoL. , ZhangX. , ShiY. , and TengT. , “LncRNA SNHG14 Contributes to the Progression of NSCLC Through miR‐206/G6PD Pathway,” Thoracic Cancer 11, no. 5 (2020): 1202–1210, 10.1111/1759-7714.13374.32153123 PMC7180566

The above article, published online on 09 March 2020 in Wiley Online Library (wileyonlinelibrary.com), has been retracted by agreement between the journal Editor‐in‐Chief, Tateaki Naito; and John Wiley & Sons Australia, Ltd. The retraction has been agreed upon following concerns raised by a third party regarding the use of an incorrect primer for the SNHG14 gene. The authors were invited to provide comments and supporting data but did not respond. Given the nature of the concern, the editors consider the results and conclusions of this article invalid. The authors did not respond to our notice of retraction.

**RETRACTION**: L. Zhao , X. Zhang , Y. Shi , and T. Teng , “LncRNA SNHG14 Contributes to the Progression of NSCLC Through miR‐206/G6PD Pathway,” Thoracic Cancer 11, no. 5 (2020): 1202–1210, 10.1111/1759-7714.13374.32153123 PMC7180566

The above article, published online on 09 March 2020 in Wiley Online Library (wileyonlinelibrary.com), has been retracted by agreement between the journal Editor‐in‐Chief, Tateaki Naito; and John Wiley & Sons Australia, Ltd. The retraction has been agreed upon following concerns raised by a third party regarding the use of an incorrect primer for the SNHG14 gene. The authors were invited to provide comments and supporting data but did not respond. Given the nature of the concern, the editors consider the results and conclusions of this article invalid. The authors did not respond to our notice of retraction.

